# Immunomodulation and iron dysregulation: exploring their roles in the pathogenesis of osteoarthritis

**DOI:** 10.1186/s12920-025-02206-4

**Published:** 2025-10-22

**Authors:** Dongmei Yang, Ye Ruan, Shengpeng Zheng, Weilong Xu, Jian Hao

**Affiliations:** 1 Orthopedics Department, Shenzhen Pingle Orthopedics Hospital(Pingshan District Hospital of Traditional Chinese Medicine), Shenzhen, Guangdong 518101 China; 2 Graduate School, The Third Clinical Medical College of Guangzhou University of Chinese Medicine, Guangzhou, Guangdong 510080 China; 3 Orthopedics Department, Guangzhou Medical University Second Affiliated Hospital, Guangdong 510220 Guangzhou, China

**Keywords:** Osteoarthritis, Iron dysregulation, Immune cell infiltration

## Abstract

**Background:**

The purpose of this study was to explore the role of immune modulation and iron metabolism disorder in the pathogenesis of osteoarthritis.

**Methods:**

We performed a systematic analysis of datasets and immune infiltration of osteoarthritis and healthy cartilage tissues, to obtain differences in genes expression and immune cell infiltration related to iron metabolism between two samples. The iron metabolism-related genes with prognostic value was screened by Lasso and Cox regression, and validated their expression in knee cartilage samples from normal and osteoarthritis patients by immunohistochemistry.

**Result:**

We obtained 494 differential genes that meet the criteria and 147 genes related to iron metabolism diseases. Many entries have been found, which Wnt signaling pathway and cholesterol metabolism may play key roles in the degradation of cartilage. The immunohistochemical staining on normal and osteoarthritis cartilage showed that iron metabolism-related genes genes were significantly higher expressed in osteoarthritis cartilage than in normal cartilage.

**Conclusions:**

The iron metabolism imbalance and immune cell subtype infiltration may be associated with the occurrence and progression of osteoarthritis. These results provide a new perspective for understanding the pathophysiology of osteoarthritis, and also offer new ideas for developing more effective treatment strategies.

**Supplementary Information:**

The online version contains supplementary material available at 10.1186/s12920-025-02206-4.

## Introduction

Osteoarthritis (OA) is a chronic disease characterized by cartilage degeneration, usually accompanied with joint pain and dysfunction [1]. Although OA is often considered a "wear and tear" disease, recent research reveals a more complex story, emphasizing the role involvement of iron metabolism and the infiltration of immune cells in its pathogenesis [2, 3]. The core mechanism appears to involve the accumulation of iron-dependent reactive oxygen species (ROS) and the ensuing inflammatory response, both of which significantly impact the homeostasis of chondrocytes [4, 5].

Iron metabolism, crucial for sustaining vital physiological functions, is involved in human DNA synthesis, mitochondrial respiration, and cell growth and death, it also contributes to host defense and cellular signal transduction [6, 7]. In inflammatory diseases, iron balance may be severely disturbed [8]. Inflammatory factors can directly upregulate hepcidin (a key role in iron metabolism), affecting the innate or adaptive immune system, and participate in disease regulation [9]. Animal research indicates that the use of hepcidin inhibitors can decrease iron levels in macrophages and mitigate inflammatory responses associated with infectious diseases [10, 11]. Iron overload or iron deficiency can also disrupt the proliferation and activation of T and B cells, intervening in the human body through ROS or by affecting mitochondrial function [12]. This is most evident in high iron conditions, such as hereditary hemochromatosis (HH), when iron accumulation leads to arthritis, cirrhosis or cardiomyopathy [13, 14]. Increasing evidence suggests that abnormally high iron status is related to OA phenotypes, including progressive cartilage degeneration, trabecular structure and biomechanical changes, persistent arthritis, proliferative synovitis and synovial folds, etc. [15–17]. OA is usually associated with chronic low-grade inflammatory state, at the same time, improper distribution and utilization of iron may exacerbate inflammation [18]. Therefore, this has sparked a renewed interest in assessing the impact of iron metabolism and inflammatory infiltration within the context of osteoarthritis.

Immune cell infiltration plays a vital role in OA joint inflammation. Notably, M1 macrophages, often referred to as pro-inflammatory, secrete a variety of cytokines such as TNF-α, IL-1β, and IL-6, which promote cartilage degradation and exacerbate joint inflammation [19, 20]. These macrophages are typically activated by pathogen-associated molecular patterns (PAMPs) and damage-associated molecular patterns (DAMPs), which are prevalent in OA-affected tissues [21]. In contrast, M2 macrophages are considered anti-inflammatory and play a role in tissue repair and resolution of inflammation by secreting cytokines like IL-10 and TGF-β [22, 23]. Iron metabolism has a profound impact on macrophage polarization, may influencing both M1 and M2 macrophages in the context of osteoarthritis (OA) [24]. Under inflammatory conditions, iron is sequestered within macrophages to restrict its availability to pathogens, a phenomenon referred to as'anemia of inflammation [25]. In OA, however, this sequestration can lead to a state of iron overload in the joint, which contributes to oxidative stress and exacerbates inflammation. High levels of intracellular iron can promote M1 macrophage polarization by enhancing the production of reactive oxygen species (ROS), which in turn stimulates inflammatory pathways such as NF-κB signaling [26, 27]. On the other hand, iron deficiency can impair M2 macrophage function, reducing tissue repair and exacerbating cartilage damage [28]. Thus, the iron imblance in OA may disrupt the equilibrium M1 and M2 macrophages, fostering chronic inflammation and impeding tissue repair. This article aims to delve into the intricate interplay between immune cell subtypes, iron metabolism, and osteoarthritis (OA), offering a new insight into understanding the pathogenesis of OA.

## Materials & methods

### Obtain and merge three disease datasets for differential analysis

We downloaded three datasets from the GEO database that contained OA and healthy samples, including GSE51588, GSE55235 and GSE55457. The inclusion of these three datasets was driven by their complementary features. While GSE55457 and GSE55235 offer large, multi-center datasets with consistent platforms for robust comparative analysis, GSE51588 adds diversity by including data from a different platform (Agilent microarray) and provides additional validation through qRT-PCR and immunohistochemical staining. For the merging of multiple datasets, we first used the R package inSilicoMerging to merge the datasets, and further used the method of Johnson WE et al. to remove batch effects. The parameters encompass the utilization of the ComBat algorithm with the subsequent configurations: a significance threshold of 0.05, empirical Bayes modification, and the incorporation of covariates such as age and gender to address potential confounding variables [29]. Limma is a differential expression screening method based on generalized linear models [30]. Here we used the R package limma (version 3.40.6) for differential analysis, to obtain differentially expressed genes between different comparison groups and control groups. Specifically, for the expression profile datasets we obtained, we used the lmFit function to perform multiple linear regression, further used the eBays function to compute moderated t-statistics, moderated F-statistic, and log-odds of differential expression by empirical Bayes moderation of the standard errors towards a common value, and finally obtained the differential significance of each gene. For differential expression analysis, we employed a threshold of *p*-value < 0.05 and a fold change of ≥ 2.

### Collect genes related to iron metabolism and mine potential disease genes

To find out the genes related to iron metabolism and their potential role in disease, we searched the Genecards database with the keyword “Iron metabolism” and intersected the results with the differentially expressed genes obtained from the previous analysis. To explore the underlying biological pathways of these genes, we performed enrichment analysis on the intersected genes. We used the clusterProfiler and org.Hs.eg.db R packages to perform GO and KEGG enrichment analyses [31, 32]. Through GO/KEGG analyses, we computed the z-score of each entry using the molecular logFC, to determine if the entries were positively (z-score > 0) or negatively regulated (z-score < 0). The GOplot package facilitated z-score calculations. We also performed Gene Set Enrichment Analysis (GSEA) using the clusterProfiler package after converting the IDs of the molecules in the input data. We used the MSigDB Collections as the gene set database, which contains detailed descriptions of various gene sets. We selected c2.cp.all.v2022.1.Hs.symbols.gmt [All Canonical Pathways] as the reference gene set, which was obtained from the msigdbr package. We used org.Hs.eg.db package for ID conversion. We calculated the enrichment score (ES) and normalized enrichment score (NES) for each gene set using GSEA. The ES reflects the degree of enrichment of genes in a gene set at both ends of a ranked list of genes. The NES is a standardized score that takes into account the number and size of gene sets. For GO/KEGG enrichment analyses, we instituted a significance threshold of p-value < 0.05. For GSEA, we utilized a false discovery rate (FDR) threshold of < 0.25. We used human (Homo sapiens) as the species for all analyses.

In addition, we performed Gene Set Variation Analysis (GSVA) to extract gene set features from the gene expression matrix. GSVA can be used to evaluate the activity changes of gene sets in different samples, or to perform subsequent differential analysis or clustering analysis [33]. The principle of GSVA is to rank the genes in each sample according to their cumulative density distribution in the gene expression matrix, and calculate a rank statistic similar to Kolmogorov–Smirnov test for each gene set, reflecting the enrichment degree of that gene set in that sample.

### Protein network and Metascape

To analyze the interactions and functions of genes related to iron metabolism in the protein network, we used two online databases: STRING and Metascape. STRING is a database that provides information on protein–protein interactions, integrating data from various sources and assigning a confidence score to each interaction. We used STRING to construct and visualize the position and relationship of our gene set in the protein network. Metascape is a database that provides functional annotation and interpretation services, integrating data from multiple sources and supporting various functional analyses. We used Metascape to perform GO enrichment and KEGG pathway analysis, to explore the biological significance and functional modules of our gene set. We used human (Homo sapiens) as the species for all analyses.

### Immune infiltration

To analyze the immune infiltration differences between OA and normal tissues, we used three different immune infiltration algorithms to calculate the infiltration scores of immune cells. One of the algorithms was CIBERSORT, which is a method based on support vector regression that can estimate the relative proportions of different types of immune cells in tissues using gene expression data. We used the marker genes of 22 types of immune cells provided by the CIBERSORTx website to perform CIBERSORT analysis. The website provided an R script to execute the CIBERSORT algorithm. We used human (Homo sapiens) as the species for the analysis [34, 35].

Another immune infiltration algorithm is ssGSEA, which is a single-sample gene set enrichment analysis method that can calculate the activity level of each gene set in each sample [33]. We used the ssGSEA algorithm provided by the R package-GSVA, and used the marker genes of 24 types of immune cells provided by the Immunity article to calculate the immune infiltration of the uploaded data [36]. These marker genes can reflect the relative abundance and functional status of different types of immune cells in tissues. The specific 24 types of immune cells and their marker genes can be found in the corresponding reference.

### LASSO and COX

To select the genes related to iron metabolism that have prognostic value in disease, we used Lasso and Cox regression methods. Lasso is a linear regression method that uses L1 regularization, which can make some of the learned feature weights zero, thus achieving sparsity and feature selection. We used the glmnet package to perform Lasso analysis on the cleaned data, obtaining the variable lambda value, maximum likelihood or C index, and visualizing the data. Lasso can be used as a variable selection method. If there are still many variables selected, we can further perform multivariate Cox regression on these selected variables to construct a Cox model. Therefore, we used the survival package to perform proportional hazard assumption test and Cox regression analysis. To evaluate the diagnostic effect, we first used the pROC package to perform ROC analysis on the data, and visualized the results with ggplot2. There are two types of ROC: single gene ROC and combined gene ROC. PR curve is a curve that reflects the relationship between precision and recall. We also used the pROC package to perform PR analysis on the data. The horizontal axis X is the recall rate, also known as the true positive rate. The closer the X axis is to 1, the higher the accuracy. The vertical axis Y is called precision, and the larger the Y axis, the better the accuracy.

To show the effect of the diagnostic model more intuitively, we also used methods such as Nomogram, calibration curve and decision curve. Nomogram is a graphical tool based on multivariate regression analysis, which can integrate multiple prediction indicators and draw them on the same plane according to a certain proportion, thus expressing the relationship between each prediction variable in the prediction model. We cleaned the data and used the glm function to construct a binary Logistic model, and used the rms package to construct a Nomogram related model and visualize it. Calibration curve is used to depict the difference between model prediction probability and actual probability, and evaluate model fit. We used the calibrate function in rms package to draw calibration curve and calculate root mean square error (RMSE) between Apparent curve (prediction curve) and Bias-corrected curve (calibration curve). Decision curve is used to describe how net benefit value changes with risk probability threshold when intervention is performed according to model prediction value. We used the dca function in rmda package to draw decision curve and calculate net benefit of each model at different thresholds.

### Specimens collection and Immunohistochemistry

OA and normal knee cartilage samples of knee were collected from patients who underwent total knee replacement (TKA) at Shenzhen Pingle Orthopedics Hospital (Pingshan District Hospital of Traditional Chinese Medicine). The Shenzhen Pingle Orthopedics Hospital(Pingshan District Hospital of Traditional Chinese Medicine) granted Ethical approval to carry out our study within its facilities(Ethical Application Ref: AF-HEC-034–01.1). We examined a total of 30 specimens in our immunohistochemistry investigations. Each specimen was meticulously chosen to guarantee a representative spectrum of the conditions under scrutiny. We incorporated both positive and negative controls in our experiments. Positive controls comprised tissues recognized to express the target antigen, while negative controls encompassed tissues that do not express the antigen, thereby ensuring the specificity of the staining. Among them, medial was OA tissue and lateral was normal tissue. The samples were fixed with 4% formaldehyde immediately after surgery and then embedded in paraffin. The sections were 5 microns thick and stained with hematoxylin–eosin after light microscopy observation.

Immunohistochemistry experiments used the following antibodies: anti-HIBCH (ab153826), anti-TLR7 (ab24184), anti-ABH63 (ab183739), anti-AZU1 (ab181989) and anti-ORM1 (ab134042). The sources, concentrations and incubation times of the antibodies are shown in Supplementary Table 8. The sections were antigen-retrieved, blocked with peroxide and protein, incubated with primary antibody overnight at 4 °C, then incubated with biotin-labeled secondary antibody for 1 h, and finally detected the signal by ABC method. The staining results were visualized with DAB and scored semi-quantitatively. Statistical analysis was performed using graphpad software. Data are expressed as mean ± standard deviation. Differences between groups were analyzed by t-test. P values less than 0.05 were considered statistically significant.

## Results

### Perform differential gene analysis after removing the batch effect

**Fig. 1:**
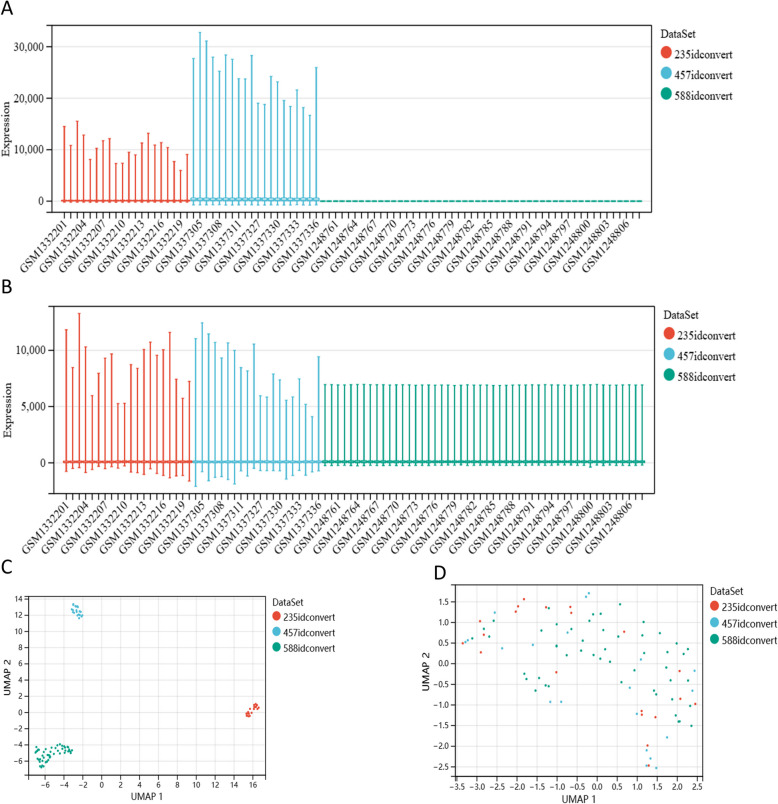
Batch effect removal of gene expression data from three datasets

**Fig. 2:**
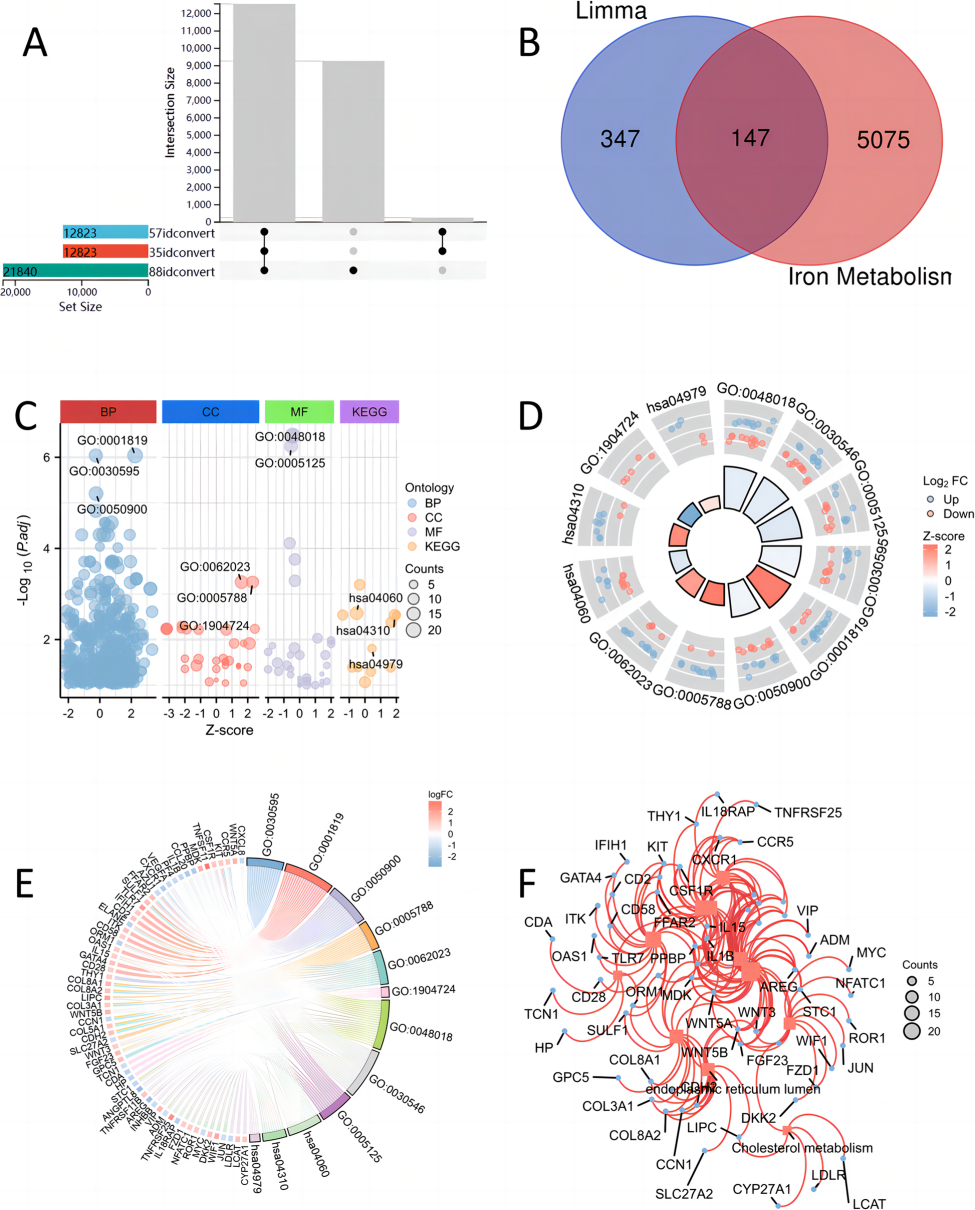
Differential gene expression and functional enrichment analysis of genes related to iron metabolism diseases in OA and healthy samples

GSE51588 contains 50 samples, GSE55235 contains 20 samples, and GSE55457 contains 20 samples (Supplementary Table 1). From the boxplot, we can observe that the sample distribution of each dataset is very different before removing the batch effect, indicating that there is a batch effect (Fig. 1A). After removing the batch effect, the data distribution of each dataset tends to be consistent, and the median is on a line (Fig. 1B). From the UMAP plot, we can observe that the samples of each dataset cluster together before removing the batch effect, indicating that there is a batch effect (Fig. 1C). After removing the batch effect, the samples of each dataset cluster and interweave with each other, indicating that the batch effect is well removed (Fig. 1D). Finally, we obtained an expression data containing 90 samples, including 60 OA samples and 30 healthy samples. After Limma differential gene analysis, we screened according to the threshold of |logFC|> 1 & adj.P.Val < 0.05, and finally obtained 494 differential genes that meet the criteria (Fig. 2A and Supplementary Table 2).

### Obtain the genes related to iron metabolism diseases and perform multiple enrichment analyses

**Fig. 3:**
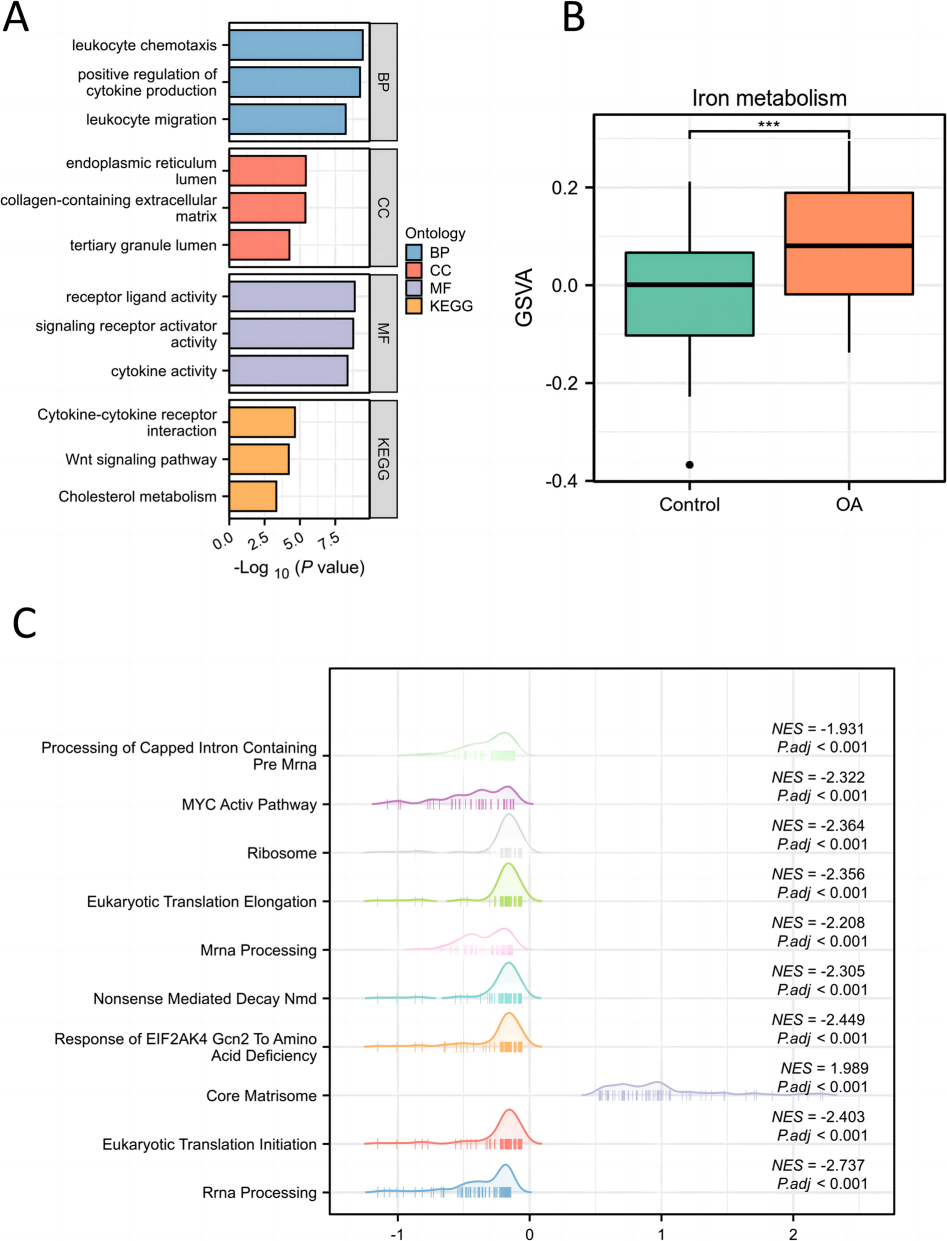
GSEA and GSVA of genes related to iron metabolism diseases in OA and healthy samples

We obtained 5222 genes related to iron metabolism from Genecards (Supplementary Table 3). After carefully correlating the data with the differentially expressed genes identified through our comprehensive analysis, we successfully identified a total of 147 genes that are intricately linked to various iron metabolism-related diseases. This discovery underscores their potential importance in elucidating the underlying mechanisms of these conditions (Fig. 2B and Supplementary Table 4). Next, we performed GO/KEGG enrichment analysis on the 147 genes (Supplementary Table 5). The analysis revealed several key findings that underscore the link between iron metabolism and osteoarthritis (OA). Within the Biological Process (BP) category, prominent terms encompassed leukocyte chemotaxis, positive regulation of cytokine production, and leukocyte migration. In terms of Cellular Components (CC), significant entries highlighted the endoplasmic reticulum lumen, collagen-rich extracellular matrix, and the lumen of tertiary granules. For Molecular Function (MF), the most enriched terms were receptor-ligand activity, signal receptor activation activity, and cytokine activity. Furthermore, KEGG pathways analysis pinpointed essential pathways such as cytokine-cytokine receptor interaction, the Wnt signaling pathway, and cholesterol metabolism, which further reinforce the association between iron metabolism and the pathogenesis of OA (Figs. 2C-F and 3A).

These results imply that leukocyte activity and migration, cytokine production and interaction, and Wnt signaling pathway may play key roles in the inflammatory response and cartilage degradation in osteoarthritis. In addition, changes in cholesterol metabolism may be related to the metabolic etiology of the disease. Overall, these biological processes and pathways may have important impacts on the occurrence and progression of osteoarthritis.

In the GSEA results, we obtained a large number of key biological processes related to gene expression and protein synthesis, such as REACTOME RRNA PROCESSING, REACTOME EUKARYOTIC TRANSLATION INITIATION, and NABA CORE MATRISOME. These entries reveal that these processes may play important roles in the disease progression of osteoarthritis (OA). Ribosomal RNA processing and protein translation regulation are essential for cell growth and differentiation, which may be especially critical for chondrocyte function and cartilage tissue reconstruction. Cell response to amino acid deficiency and nonsense-mediated decay mechanism may be related to chondrocyte survival and adaptability in the disease environment. Moreover, MYC gene activation pathway and extracellular matrix composition may be associated with inflammation and cartilage degradation in OA (Fig. 3C and Supplementary Table 6).

In general, these biological processes may play key roles in the disease progression of osteoarthritis, affecting the structure and function of cartilage, thereby aggravating or alleviating the severity of the disease. Finally, we performed GSVA based on these 147 genes related to iron metabolism diseases (Fig. 3B and Supplementary Table 7). The process finally obtained a biological score to evaluate iron metabolism, which can be found that compared with normal tissues, this iron metabolism-related score was significantly increased in OA disease.

### Protein–protein interaction and Metascape

**Fig. 4:**
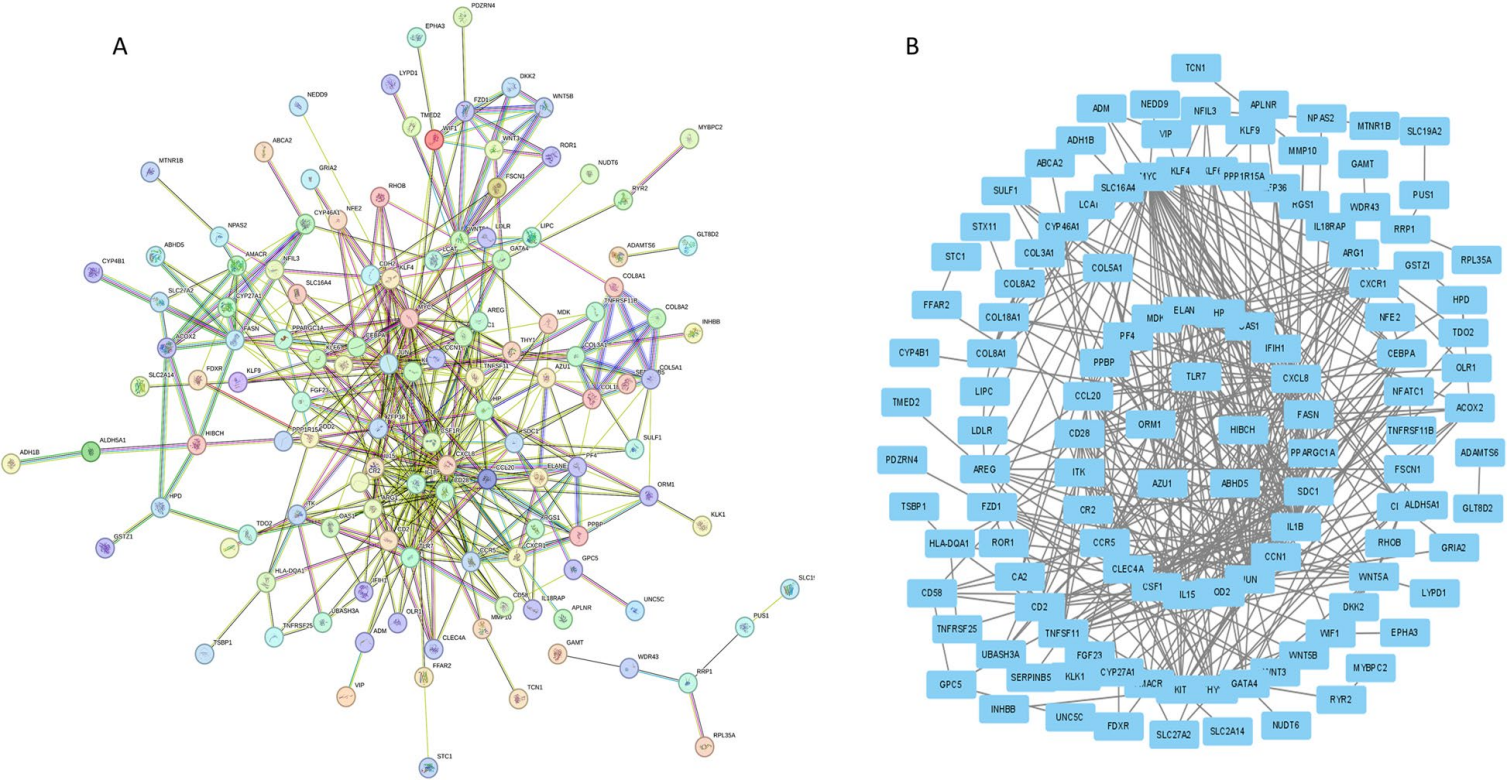
Iron metabolism-related PPI network of the 147 genes

**Fig. 5:**
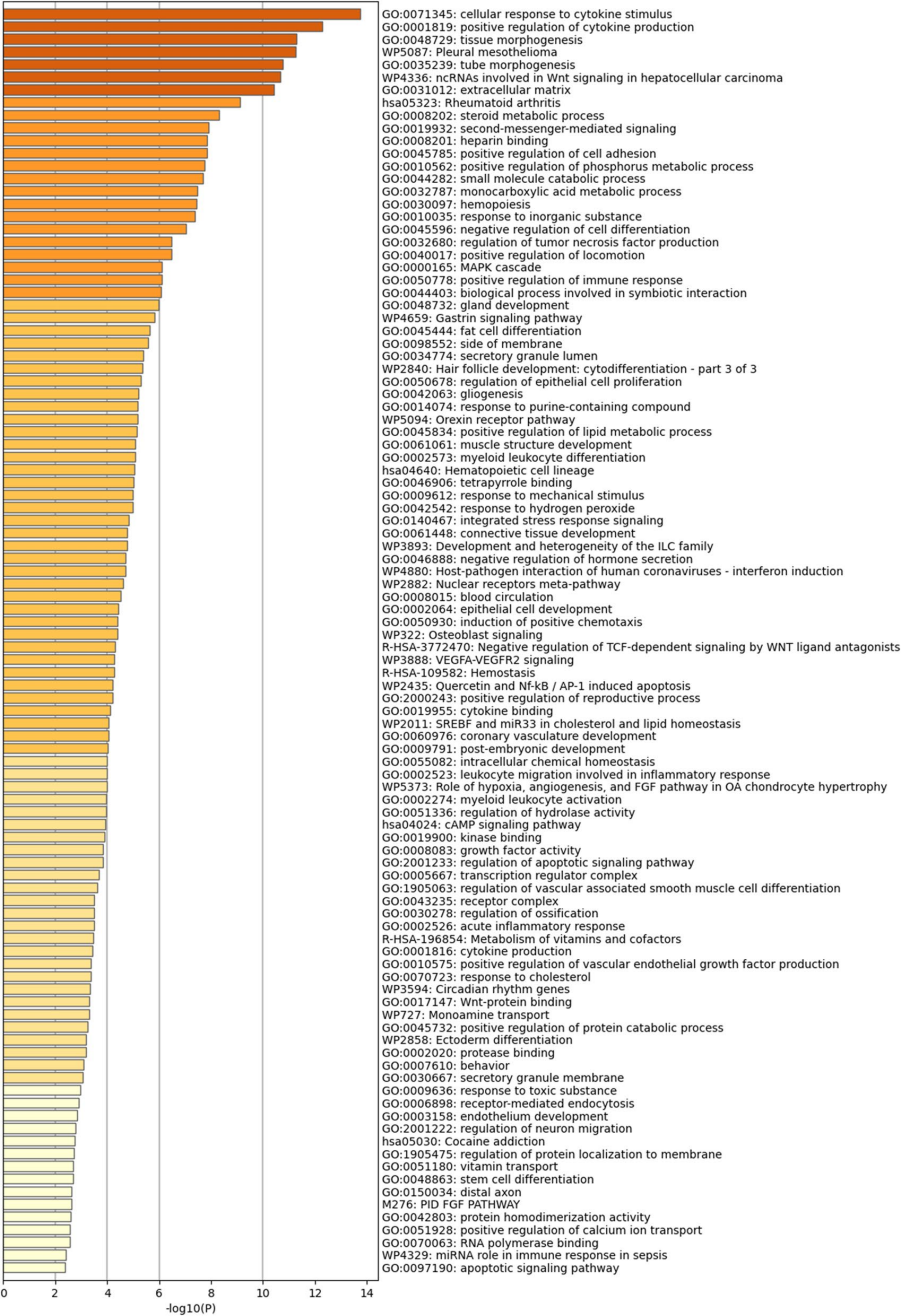
Enrichment analysis of biological pathways related to iron metabolism in OA and healthy samples

We input these 147 genes related to iron metabolism diseases into the string database and obtained an iron metabolism-related PPI. We found that these genes have strong interactions with each other. It has been shown that a small fraction of highly connected regions (subnetworks) in PPI networks are more likely to be involved in biological regulation, while those lightly connected nodes do not play a key role in the integrity of the entire network (Fig. 4A and B and Supplementary Table 9). For this purpose, we used cytoscape software and its plugin MCODE to mine the entire iron metabolism-related PPI and finally obtained several key subnetworks. In Metascape, we obtained biological pathways such as cellular response to cytokine stimulus, inflammatory response, signaling receptor regulator activity, and NABA MATRISOME ASSOCIATED (Fig. 5 and Supplementary Table 10). These results mainly involve inflammation, cytokine production and response, as well as cell migration and tissue morphogenesis. Inflammation and leukocyte chemotaxis and migration are key in OA, as they are associated with joint inflammation and cartilage degradation. In particular, cell response to cytokines, positive regulation of cytokine activity, receptor ligand activity, and signal receptor activation activity are related to the role of cytokines in OA, which may promote inflammation and cartilage degradation.

In Metascape, the analysis unveiled biological pathways such as cellular response to cytokine stimulus, inflammatory response, signaling receptor regulator activity, and NABA MATRISOME ASSOCIATED. These pathways predominantly encompass inflammation, cytokine production and response, cell migration, and tissue morphogenesis, all of which are essential to OA development and progression. Pathways associated with cellular response to cytokines, positive modulation of cytokine activity, receptor ligand activity, and signal receptor activation activity underscore the significance of cytokines in OA. These cytokines are recognized for fostering joint inflammation and cartilage degradation, fundamental pathological characteristics of OA. Terms such as NABA MATRISOME ASSOCIATED and NABA SECRETED FACTORS accentuate the criticality of ECM integrity in preserving cartilage structure and function. Tissue morphogenesis pathways imply a role in cartilage reconstruction and repair, which are vital for both the progression and management of OA. Leukocyte chemotaxis and migration are pivotal to joint inflammation and immune responses in OA, further exacerbating cartilage degradation and disease advancement. While the dataset also indicated pathways like Pleural Mesothelioma, this seems unrelated to OA pathogenesis and may reflect specific genes or pathways unique to the dataset rather than a direct link to OA. Overall, the identified subnetworks highlight the central roles of inflammation, cytokine signaling, cell migration, and ECM integrity in the emergence and progression of OA, offering a deeper insight into its molecular mechanisms and potential therapeutic targets.

### Immune infiltration

**Fig. 6:**
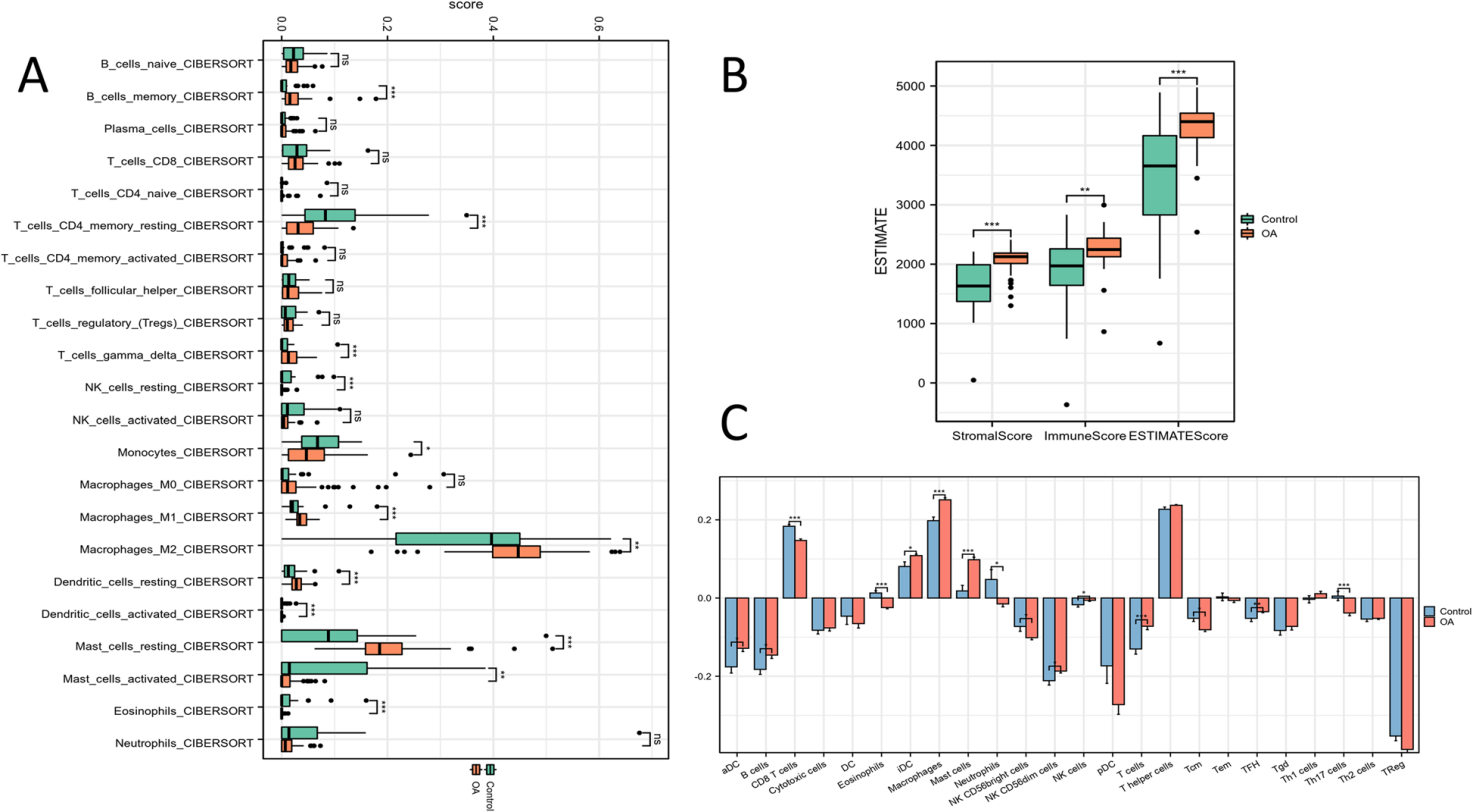
Immune cell infiltration analysis of OA and healthy samples

From the results calculated by the CIBERSORT algorithm, it can be seen that B cells memory and T cells gamma delta are significantly highly infiltrated in OA, but CD4T memory resting and NK cells resting show the opposite trend. In addition, monocytes show lower infiltration levels in OA, but polarized M1 and M2 type Macrophages are significantly highly infiltrated. Moreover, Dendritic cells and Mast cells are more in the resting state than activated. Finally, Eosinophils are significantly low infiltrated in OA, while Neutrophils show no significant changes (Fig. 6A and Supplementary Table 11). According to the ESTIMATE algorithm to calculate the matrix and immune scores, it is found that both of them are significantly increased, indicating that in OA, there is stronger matrix cell and immune cell infiltration (Fig. 6B and Supplementary Table 12). ssGSEA supplemented that CD8T cells also showed a significant low infiltration level in OA. At the same time, it also suggested that in OA, immature Dendritic cells and Msat cells were significantly increased. This implies that although we have a strong immune cell infiltration level in OA, a large part of the immune cells are in an immature or resting state with no normal function. In addition, ssGSEA supplemented the infiltration status of different T cell subtypes, Tcm and Th17 infiltration levels were significantly increased, while TFH was completely opposite (Fig. 6C and Supplementary Table 13). The immune infiltration analysis, facilitated by the CIBERSORT algorithm, disclosed several notable shifts in immune cell infiltration patterns associated with osteoarthritis (OA). Memory B cells and gamma delta T cells demonstrated a significant increase in infiltration, while resting CD4 T cells and natural killer (NK) cells exhibited a reduction in infiltration levels. The presence of highly infiltrated polarized M1 and M2 macrophages suggests an enhanced macrophage engagement in OA pathology. Moreover, dendritic cells and mast cells predominantly remained in a resting state, eosinophils showed significantly reduced infiltration, and neutrophil infiltration remained relatively stable.

The ssGSEA analysis offered additional insights, emphasizing a notable decrease in CD8 T cell infiltration alongside an increase in immature dendritic cells and mast cells, indicating a prevalence of immune cells in immature or resting states. Moreover, ssGSEA uncovered subtype-specific alterations in T cell populations, with central memory T cells (Tcm) and Th17 cells displaying elevated infiltration, in stark contrast to a significant reduction in follicular helper T cells (Tfh).

These findings highlight the intricate immune microenvironment in osteoarthritis (OA), characterized by increased immune cell infiltration and a concurrent shift in their functional states. The elevated presence of macrophages and Th17 cells is consistent with the inflammatory profile of OA, given their established roles in fostering pro-inflammatory cytokine secretion and joint tissue damage. On the flip side, the diminished presence of functional CD8 T cells and the immature or resting state of other immune cells may indicate a compromised immune resolution process, which in turn, contributes to the chronic inflammation and tissue degradation that are hallmarks of OA. These observations underscore the dualistic role of immune cells in both sustaining inflammation and failing to resolve it efficiently within the context of OA.

### Lasso and cox

**Fig. 7:**
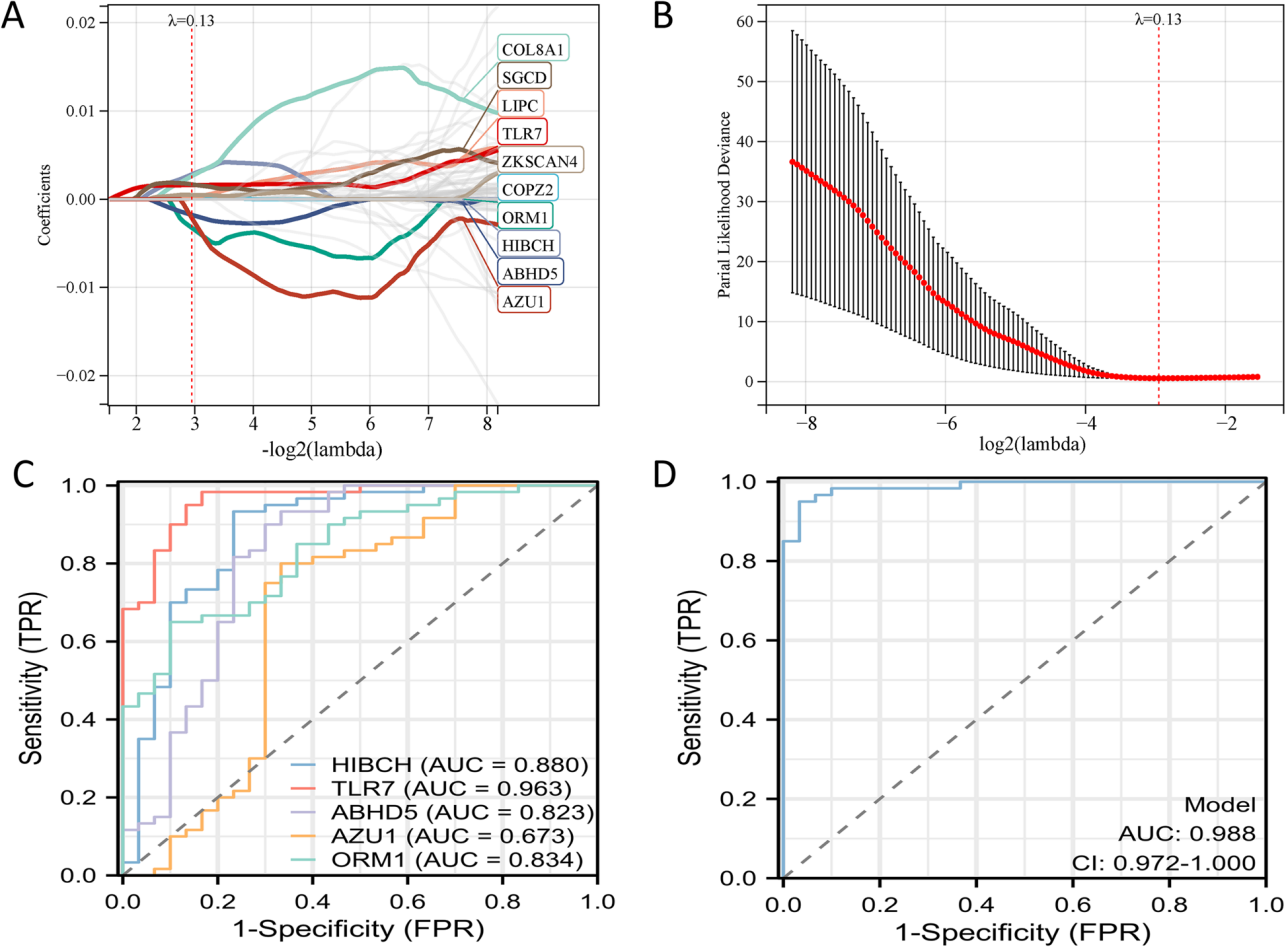
Screening and evaluation of key metabolic genes related to iron metabolism in OA and healthy samples

In order to mine the key metabolic genes of iron metabolism, we combined Lasso and COX regression analysis for screening. First, after screening by Lasso regression analysis, we obtained ten potential genes, and further screened by COX regression, and finally obtained five genes related to iron metabolism diseases. In order to evaluate the value of these five genes for diagnosing OA, we constructed models such as ROC and PR curves to discuss the effects. In the process of constructing diagnostic models with genes identified via LASSO and Cox regression analyses, it was noted that the incorporation of the HIBCH variable led to the model's predictive values clustering at the extremes of 0 or 1. This observation indicated that HIBCH might be introducing bias or diminishing the model's overall predictive flexibility. To mitigate this, we opted to exclude the HIBCH variable, which yielded a more balanced and robust model with enhanced predictive capabilities. This refinement ensures that the model more faithfully represents the diagnostic potential of the remaining genes for osteoarthritis (OA) (Fig. 7A-D and Supplementary Fig. 1).

### Immunohistochemistry

**Fig. 8:**
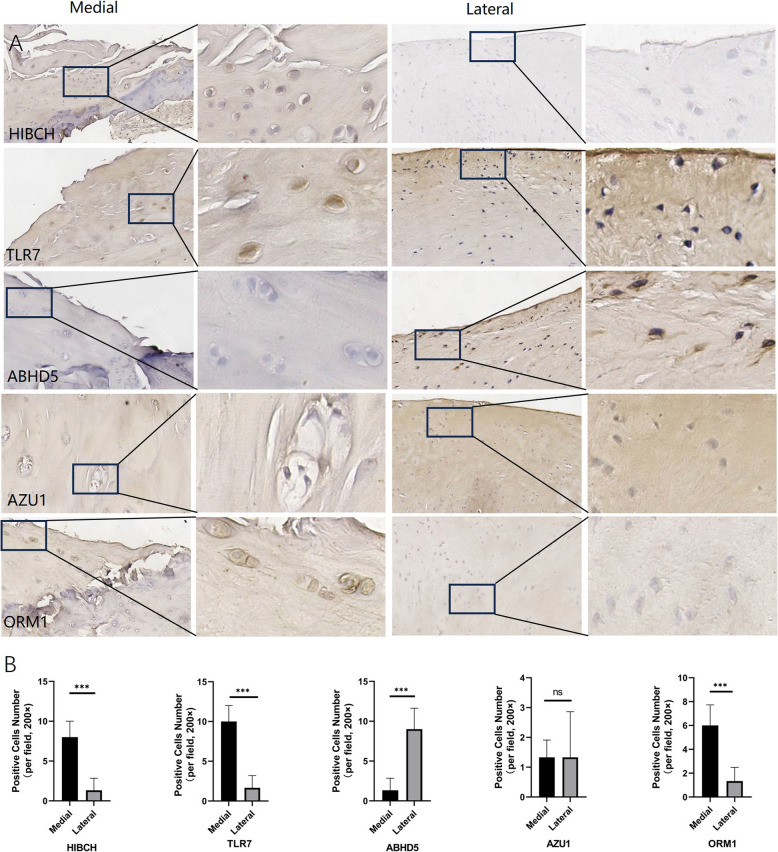
Immunohistochemical staining of iron metabolism-related genes in normal and osteoarthritis cartilage sections

To verify the expression of iron metabolism-related genes in cartilage, we performed immunohistochemical staining on normal and osteoarthritis cartilage sections. We selected five iron metabolism-related genes: HBCh, TLR7, ABH63, AZU1 and ORM1, and stained them with corresponding primary and secondary antibodies, and visualized them with DAB (Fig. 8 A and B). The results showed that HBCh, TLR7 and ORM1 genes were significantly higher expressed in osteoarthritis cartilage than in normal cartilage, and mainly distributed in the cytoplasm and nucleus of chondrocytes (Fig. 7), while ABH63 was opposite, and AZU1 had no difference between OA and normal tissues. The immunohistochemistry findings served as a crucial validation for the computational gene expression analysis, corroborating the results for the majority of genes and pinpointing a few discrepancies. Notably, the immunohistochemical staining revealed markedly elevated expression levels of HBCh, TLR7, and ORM1 in osteoarthritis (OA) cartilage when compared to normal cartilage, which is in concordance with the in-silico analysis. These genes were predominantly detected in the cytoplasm and nucleus of chondrocytes, further substantiating their involvement in the pathological alterations characteristic of OA.

On the other hand, ABH63 showed reduced expression in OA cartilage, aligning with the computational predictions and further validating the reliability of the in-silico analysis. However, AZU1 did not exhibit a significant difference between OA and normal tissues, contradicting the computational findings. This inconsistency might indicate potential limitations within the computational data or suggest that post-transcriptional regulation, protein stability, or localization factors, which are not reflected in gene expression analyses.

Overall, the immunohistochemical results largely support the computational findings, enhancing the credibility of the study's conclusions.

## Discussion

Osteoarthritis is a common degenerative joint disease, which involves multiple factors in its occurrence and development, among which iron metabolism and immune response may be important links. This study analyzed the expression of iron metabolism-related genes and the infiltration of immune cell subtypes, and explored the relationship between iron metabolism, immune cell infiltration and osteoarthritis, providing a new perspective for understanding the pathogenesis and progression of this disease.

We found that iron metabolism-related genes were abnormally expressed in osteoarthritis, reflecting the imbalance of iron homeostasis and uneven distribution of iron. This may be related to the increase of inflammation and oxidative stress, and may also affect the structure and function of cartilage. Iron is an indispensable element for life, but under inflammatory conditions, iron metabolism is usually strictly regulated [37]. In osteoarthritis, inflammation can cause abnormal distribution and utilization of iron, which may lead to local or systemic imbalance of iron homeostasis [38]. Highly active inflammatory state is usually accompanied by iron “sequestration”, making it difficult for the body to effectively use it, thus affecting the normal demand of the body for iron [39, 40]. On the other hand, Iron overload induces the production of reactive oxygen species (ROS), which activates inflammatory signaling pathways such as NF-κB, leading to M1 macrophage polarization and exacerbating joint inflammation [41]. Furthermore, elevated ferritin levels in OA synovial fluid correlate with increased inflammatory cytokine production, suggesting a direct link between iron dysregulation and joint inflammation [42]. In addition, iron has important functions in chondrocytes, including the synthesis of collagen and proteoglycan [43]. Therefore, iron metabolism disorder may directly affect the structure and function of cartilage, and then affect the progression of osteoarthritis.

We also found that immune cell subtypes infiltrated differently in osteoarthritis, showing complex immune regulation mechanisms. Among them, B cells memory and T cells gamma delta increased infiltration may be related to their role in promoting inflammation and immune response; CD4T memory resting and NK cells resting decreased infiltration may be related to their role in inhibiting excessive immune response; monocytes decreased infiltration, but M1 and M2 type Macrophages increased infiltration, which may reflect the balance between M1 and M2 type Macrophages in promoting or inhibiting inflammation and repair process. Immune cells are key cell types in osteoarthritis, they can secrete a variety of cytokines, affecting cartilage degradation, synovial hyperplasia, angiogenesis and other processes.

There are complex interactions and regulation between immune cell subtypes, they are essential for maintaining joint internal environment stability [44, 45]. We observed that B cells memory and T cells gamma delta were significantly infiltrated in OA, which may be related to their important role in providing long-term immune memory, participating in early immune response and promoting inflammation. T cells gamma delta are important immune cells, they can quickly identify and kill damaged cells, while secreting a variety of cytokines, regulating the activity of other immune cells [46]. B cells memory can provide long-term immune memory, which is important for preventing disease recurrence or preventing infection [47]. On the contrary, CD4T memory resting and NK cells resting decreased infiltration in OA may reflect their regulatory role in disease progression. CD4T memory resting are highly plastic T cell subtypes, they can transform into different effector T cells according to environmental factors, such as Th1, Th2, Th17 etc. [48]. NK cells resting are part of innate immunity, they are essential for regulating inflammation and immune response [49]. These cells may be suppressed or regulated in OA to prevent excessive immune response leading to joint damage. In addition, we also observed that monocytes infiltrated lower in OA, but M1 and M2 type Macrophages infiltrated significantly increased. This is very interesting because Macrophages are key cells in joint inflammation, they can secrete various inflammatory factors, promote cartilage degradation. M1 type Macrophages have pro-inflammatory effects, while M2 type have anti-inflammatory effects, which may reflect the balance between the two in the progression of the disease [50, 51]. M1 and M2 type Macrophages balance may depend on various factors in the joint microenvironment, such as cytokines, growth factors and signaling molecules, which may come from damaged cartilage, synovium or other joint tissues [52]. At the same time, Macrophages are also highly plastic cells, capable of changing their phenotype and function according to environmental factors [53]. In OA, the same Macrophages may show M1 or M2 type characteristics at different stages or locations.

Our findings align with previous studies suggesting that iron dysregulation and immune cell infiltration play crucial roles in osteoarthritis (OA) progression [54]. And our study expands upon this knowledge by elucidating the specific impact of iron metabolism on macrophage polarization and by pinpointing potential therapeutic targets within iron-regulating pathways. Significantly, while previous studies have predominantly concentrated on systemic iron overload, our research emphasizes the localized dysregulation of iron within the joint microenvironment. This focus on local iron imbalance may pave the way for more targeted treatment strategies. The localized disruption of iron homeostasis within the OA joint environment is a pivotal factor that could lead to the development of more precise therapeutic interventions that address the fundamental causes of OA progression.

Future research should integrate proteomics and functional experiments to validate the molecular mechanisms underlying the relationship between iron metabolism and immune cell infiltration in osteoarthritis (OA). Advanced in vitro models, such as co-culture systems of chondrocytes and macrophages, can provide valuable insights into the direct effects of iron dysregulation on immune cell behavior and cartilage integrity. To establish causality and evaluate therapeutic potential, in vivo animal models should be employed. Genetic or pharmacological modulation of iron metabolism, such as using iron chelators or inducing iron overload, can be tested in transgenic or knockout mice. However, all in vivo studies must adhere to strict ethical guidelines to ensure animal welfare, including minimizing suffering through appropriate anesthesia, following the 3Rs principle (Replacement, Reduction, Refinement), and obtaining institutional ethical review board approval. Additionally, careful monitoring is necessary when performing genetic modifications such as gene knockouts to avoid unintended consequences on animal health. Ethical considerations will also extend to the potential long-term impacts of interventions, ensuring that the benefits outweigh the potential harm. Moreover, these approaches could identify therapeutic targets, such as interventions to restore iron homeostasis or modulate macrophage polarization. Targeted delivery of iron chelators or ROS inhibitors to inflamed joints may mitigate oxidative stress and cartilage damage, offering a promising strategy to slow OA progression. Together, these strategies could significantly advance our understanding of OA pathogenesis and support the development of novel treatments.

In conclusion, this study highlights the critical roles of iron metabolism dysregulation and immune modulation in the pathogenesis of osteoarthritis (OA), providing a foundation for future therapeutic strategies. These insights not only deepen our understanding of OA’s complex molecular mechanisms but also identify potential therapeutic targets, such as iron-regulating pathways and immune modulation, which could develop novel, more effective treatments. Ultimately, these efforts could pave the way for the development of more effective, targeted treatments that not only alleviate inflammation and promote cartilage repair but also address the root causes of OA, leading to improved long-term patient outcomes.

## Conclusions

This paper analyzed the differential expression of iron metabolism-related genes and the distribution of immune cell subtypes, revealing the relationship between iron metabolism, immune regulation and osteoarthritis. It found that iron metabolism disorder and immune cell subtype change may affect the development of osteoarthritis, involving inflammation, cytokine release, cell migration and cartilage structure damage. It screened for iron metabolism-related genes with prognostic significance by Lasso and Cox regression methods, and confirmed their expression levels in cartilage by immunohistochemistry methods. It provided a new perspective for understanding the pathophysiological mechanism of osteoarthritis, and also provided new ideas for developing more effective treatment strategies.

## Supplementary Information

Supplementary Material 1.

## Data Availability

Data is provided within the manuscript or supplementary information files.
