# Supplementary figures and images for: Immunomodulation and iron dysregulation: exploring their roles in the pathogenesis of osteoarthritis
Source: BMC Med Genomics. 2025 Oct 22;18:167. doi: 10.1186/s12920-025-02206-4 (PMC12542007; doi:10.1186/s12920-025-02206-4)

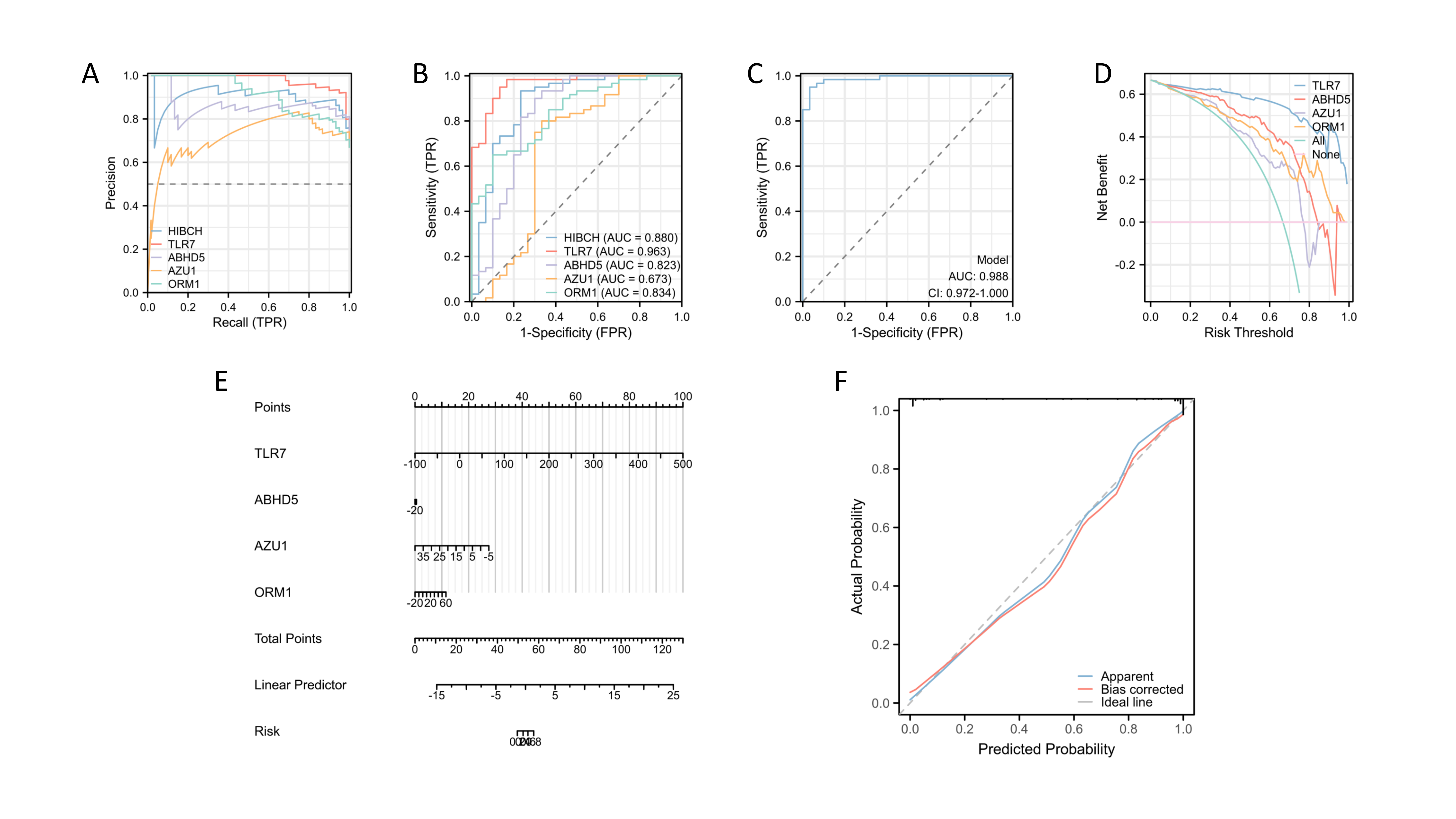

Supplement: Supplementary file 1 — Supplementary Material 1. [file 12920_2025_2206_MOESM1_ESM.zip › Supplementary/Supplementary figure 1.png]
